# The Landmark Series: Extent of Surgery for Low-Risk Differentiated Thyroid Cancer

**DOI:** 10.1245/s10434-025-17063-9

**Published:** 2025-02-26

**Authors:** Timothy M. Ullmann, Julie A. Sosa

**Affiliations:** 1 Department of Surgery, Albany Medical College, Albany, NY USA; 2 Department of Surgery, University of California San Francisco (UCSF), San Francisco, CA USA

## Abstract

The management of patients with differentiated thyroid cancers (DTCs) at low risk for disease progression or relapse after treatment remains controversial. These patients have excellent disease-specific survival. Therefore, minimizing the impact of treatments on patients’ quality of life is particularly important. For these reasons, the pendulum has swung in recent years to favor less extensive surgery toward lobectomy instead of total thyroidectomy, away from prophylactic (central compartment) lymphadenectomy, and even in some cases, omitting surgery altogether. This review discusses several of the influential studies from the past two decades that have had an impact on the management for these patients, including a shift toward more personalized care.

Thyroidectomy is the standard-of-care treatment for the majority of patients with differentiated thyroid cancer (DTC), including those considered to be at low risk for recurrent or progressive disease. Although there is no consensus on the specific criteria to define “low-risk” thyroid cancers, in this review we define low-risk thyroid cancers as papillary, follicular, or oncocytic thyroid carcinomas smaller than 4 cm and entirely confined to the thyroid (no clinical or radiographic evidence of gross extrathyroidal extension, regional or distant metastases, or local invasion). The treatment for these tumors remains controversial and continues to evolve.

In recent years, the extent of surgical resection for low-risk tumors has seen a transition from total thyroidectomy to thyroid lobectomy and away from prophylactic central neck compartment lymphadenectomy.^1^ These shifts came after key studies demonstrating equivalent survival for patients treated with thyroid lobectomy and those treated with total thyroidectomy^2,3^ and a lack of benefit from radioactive iodine (RAI) treatment for these patients.^4^ Small trials also have failed to demonstrate a benefit of prophylactic central neck dissection.^5,6^

A growing body of research has found a low rate of disease progression during active surveillance^7^ or after ablation of small low-risk cancers.^8^ Current treatment focuses on a patient-centered approach; especially when evidential equipoise exists between treatment options.^9,10^ Advances such as better understanding of the molecular mechanisms that drive aggressive tumor behavior may allow further personalization in the future.^11^

This review aims to summarize and assess some of the key studies that have influenced current guidelines regarding the surgical management of low-risk differentiated thyroid cancers in four areas: lobectomy versus total thyroidectomy, use of adjuvant radioactive iodine, prophylactic central neck dissection, and active surveillance and ablative techniques for tumors smaller than 1 cm.

## Literature Search

A literature search was performed using the EMBASE and MEDLINE databases for “extent of surgery low-risk thyroid cancer” for English language articles published from 2010 through October 2024. Results were filtered for “controlled trial,” “clinical trial,” “randomized controlled trial,” “controlled clinical trial,” or “comparative study.” Selected studies were chosen by the authors based on their relevance to the topic of this review and influence on current guidelines. Additional studies were added to the review based on their inclusion in and influence on the American Thyroid Association (ATA) guidelines for the management of differentiated thyroid cancers.^1^

## Thyroid Lobectomy Versus Total Thyroidectomy

### Recurrence and Survival

In 2007, Bilimoria et al.^12^ published an influential analysis of the American College of Surgeons’ National Cancer Database (NCDB), finding a survival advantage for total thyroidectomy over lobectomy for patients with papillary thyroid cancers (PTC) larger than 1 cm. They included patients with PTC treated between 1985 (the first year from which NCDB data are available) and 1998. The authors excluded pediatric patients, poorly differentiated tumors, and subjects missing data on extent of surgery or tumor size. The primary outcomes assessed were recurrence-free and overall survival.

The study included more than 52,000 patients, of whom approximately 83% were treated with total thyroidectomy, either upfront or in a staged fashion. The authors found an increase in unadjusted recurrence rates (9.8% vs. 7.7%) and decreased 10-year survival (97.1% vs. 98.4%) for patients treated with thyroid lobectomy versus total thyroidectomy (*P* < 0.05 for both). They then used Cox proportional hazards modeling to adjust for demographic, pathologic, and clinical confounders (Table 1).

**Table 1:** Comparison of Cox proportional hazard analyses in Bilimoria et al.^12^ and Adam et al.^2^

	Bilimoria et al. (2007)		Adam et al. (2014)	
Tumor size (cm)	1.0–2.0	2.1–4.0	1.0–2.0	2.1–4.0
Lobectomy vs. total thyroidectomy: Cox proportional HR (95% CI)	1.49 (1.02–2.17)	1.31 (1.01–1.69)	0.95 (0.79–1.13)	1.12 (0.93–1.36)
Independent variables	Age (< 45, 45–64, ≥ 65 years), sex, race (white, black, Asian, Hispanic, other), annual income (< $36,000 vs. ≥ $36,000), absence or presence of nodal metastases, absence or presence of distant metastases, RAI treatment, year of diagnosis (1985–1993 vs. 1994–1998), and hospital volume		Age (by decade), sex, race (white, black, Asian, other), annual income (< $35,000 vs. ≥ $35,000), insurance status, hospital volume (per 10 cases/year), patient comorbidities (Charlson-Deyo score 0, 1, ≥ 2), tumor multifocality, extrathyroidal extension, lymph node involvement, distant metastases, surgical margin status, and RAI treatment	

HR, hazard ratio; CI, confidence interval; RAI, radioactive iodine

Stratified analyses by tumor size showed no difference in survival based on extent of surgery for patients with tumors smaller than 1 cm, but a 24% increase in the risk of death for patients treated with thyroid lobectomy for tumors 1 to 2 cm in size and a 26% increased risk of death for patients with tumors 2 to 4 cm in size (Table 1). In line with these findings, the 2009 American Thyroid Association (ATA) guidelines recommended total thyroidectomy for all patients with DTC larger than 1 cm.^13^

These results were not without controversy, however. The reliability of the NCDB data on cancer recurrence was considered questionable, and recurrence data were subsequently removed from the database.^14^ Several other key variables were later added to the NCDB, including the presence of extrathyroidal extension, surgical margin status, and tumor multifocality. Socioeconomic, demographic, and comorbidity variables also were made more robust in subsequent editions of the database. These changes allowed for more rigorous comparison of survival after thyroid lobectomy or total thyroidectomy for patients with PTC.

In 2014, Adam et al.^2^ published their analysis of NCDB data from 1998 to 2006. They included all adult patients with PTCs between 1 and 4 cm in size and excluded patients with aggressive variant histology and those with other cancer types or treatments. Their study included almost 62,000 patients, of whom approximately 89% underwent total thyroidectomy.

Like the prior study by Bilimoria et al.,^12^ Adam et al.^2^ found an unadjusted survival advantage for patients treated with total thyroidectomy instead of thyroid lobectomy (92.9% vs. 91.4%) at 10 years. They also replicated the finding that survival decreased with increasing tumor size. However, unlike Bilimoria et al.,^12^ they were able to adjust for tumor multifocality, extrathyroidal extension, and surgical margin status, among other factors (Table 1). Using adjusted Cox proportional hazards ratios, they found no difference in survival between total thyroidectomy and thyroid lobectomy for patients with tumors 1 to 4 cm in size (hazard ratio [HR], 0.96; 95% confidence interval [CI], 0.84–1.09; *P* = 0.54; Table 1). In 2016, after the publication of this and other similar studies, the ATA changed guideline recommendations to state that either total thyroidectomy or thyroid lobectomy is an acceptable operation for low-risk DTC.^1^

### Quality of Life

Patient-reported outcomes are becoming increasingly important tools to inform surgical decision-making, particularly for low-risk elective surgeries such as thyroidectomy and cases in which the overall quality of the data is low.^9^ In 2022, Chen et al.^15^ published a cohort study involving 1060 adult Chinese patients who underwent either thyroid lobectomy or total thyroidectomy for low- or intermediate-risk thyroid cancers during a 1-year period from October 2018 through September 2019. The participants filled out a patient satisfaction questionnaire and three standardized questionnaires designed to assess health care-related quality of life (HRQOL). The patients were surveyed 1, 3, 6, and 12 months postoperatively.

Most of the participants (828 patients, 78.1%) were women with a median age of 38 years. Slightly more than half of the patients (53.1%) were treated with thyroid lobectomy.^15^ The survey response rate varied by time point, but was above 70% for all time points. The patients treated with total thyroidectomy had higher-risk disease and were more likely to undergo RAI and lymphadenectomy. The baseline HRQOL dimensions were similar between the groups, but 1 month after surgery, the patients who had undergone total thyroidectomy reported more anxiety, depression, fatigue, pain, voice change, tingling, and other symptoms, and correspondingly worse psychosocial function. However, these differences disappeared by 6 months postoperatively. The authors adjusted for preoperative HRQOL, marital status, tumor size, extent of lymph node dissection, and RAI treatment and found the same patterns in self-reported outcomes. The authors concluded that there is no HRQOL advantage to either lobectomy or total thyroidectomy because long-term (1 year) HRQOL indices are similar for patients treated with the two operations.^15^ However, it is the opinion of the authors of this review that improved short-term HRQOL is an important advantage of thyroid lobectomy over total thyroidectomy when other factors are equivalent. Additionally, thyroid lobectomy has a lower complication rate, even when performed by high-volume surgeons.^16^ Notably, other studies have shown that patients often have not been offered lobectomy for low-risk cancers,^17^ despite the fact that many would prefer it.^17,18^

### Other Factors

In addition to oncologic outcomes, other indications for thyroidectomy such as the presence of concerning contralateral nodules, hyperthyroidism specifically, Graves disease or a toxic multinodular goiter, family history or the presence of a known genetic predisposition, and exposure to carcinogens such as ionizing radiation may favor total thyroidectomy over thyroid lobectomy.^1^ Whether certain genetic alterations predispose patients to more aggressive disease and therefore should push patients and surgeons toward total thyroidectomy remains controversial. In particular, the loss of tumor suppressor gene function, such as *TP53,*^11,19^ and combinations of genetic mutations, such as *BRAF*^*V600E*^ with *TERT* promoter mutations^20,21^ or *BRAF*^*V600E*^ with mutations in the PI3K pathway,^22^ have been associated with more aggressive disease.

A recent study using ThyroSeq version 3 found that its Cancer Risk Classifier molecular risks groups were able to accurately predict the risk of recurrent disease after surgery based on molecular testing of fine-needle aspirate biopsy samples.^23^ Molecular testing also can identify targetable mutations for patients with advanced disease.^11^ It is possible that in the future, tumor genotype may play an important role in determining the optimal extent of surgery for otherwise low-risk thyroid cancer patients.

### Radioactive Iodine

The rationale for using total thyroidectomy to treat unilateral non-metastatic thyroid cancers typically is to allow for effective remnant ablation with RAI. However, RAI is used less and less frequently for low-risk DTC,^24^ and it is no longer routinely recommended by the ATA for these patients.^1^ This recommendation was based largely on observational studies and metanalyses.^1^ In a cohort of 1298 low-risk DTC patients treated between 1975 and 2005 in France with a median follow-up period of 10 years, there was no difference in risk of recurrence or death based on whether the patients were treated with surgery followed by RAI or surgery alone.^25^ A more contemporary metanalysis found the same result: for low-risk differentiated thyroid cancer patients, RAI was not associated with lower recurrence rates.^26^

Recently, the ESTIMABL2 trial, completed in France, compared 3-year outcomes for patients with low-risk differentiated thyroid cancers randomized to total thyroidectomy with or without adjuvant RAI.^4^ The study included adult patients with multifocal T1a (all tumors smaller than 1 cm and the sum of the largest diameters of each tumor totaling < 2 cm) or unifocal T1b (< 2 cm) tumors with N0 or Nx pathology and no aggressive features. The primary end point assessed was “event-free” survival. Definitions of the events are listed in Table 2 and represent functional, biochemical, or structural evidence of recurrent thyroid cancer.

**Table 2:** Definition of “event” used in the ESTIMABL2 trial^4^

Event type	Radioiodine treatment group	No-radioiodine group
Functional	Foci of iodine uptake outside the thyroid bed on post-ablation scan prompting either additional RAI treatment or surgery	N/A
Biologic: negative TgAb	Stimulated Tg level > 5 ng/mL, or unstimulated Tg level > 5 ng/mL, or unstimulated Tg level > 1 ng/mL on two consecutive tests 6 months apart	Unstimulated Tg level > 5 ng/mL or unstimulated Tg level > 2 ng/mL on two consecutive tests 6 months apart
Biologic: positive TgAb	TgAb titers above the upper limit of normal or an increase in TgAb titers > 50% in two consecutive tests 6 months apart	TgAb titers above the upper limit of normal or an increase in TgAb titers > 50% in two consecutive tests 6 months apart
Structural	Abnormal lymph node(s) or thyroid bed mass(es) on ultrasound and either abnormal FNA cytology, Tg washout levels > 10 ng/mL on FNA, or concurrent biologic event	Abnormal lymph node(s) or thyroid bed mass(es) on ultrasound and either abnormal FNA cytology, Tg washout levels > 10 ng/mL on FNA, or concurrent biologic event

RAI, radioactive iodine; N/A, not applicable; TgAb, thyroglobulin antibodies; Tg, thyroglobulin; FNA, fine-needle aspirate

The patients were randomized 2 to 5 months postoperatively after total thyroidectomy with or without central neck lymph node dissection. In this trial, 776 patients were randomized, and 3-year follow-up data were available for 730 (94.1%) of these patients. The patients in the RAI group were treated with 30 mCi of I-131 24 h after administration of recombinant human thyrotropin (rhTSH). Levels of Tg and TgAb were measured 10 months after randomization and annually thereafter. In the RAI group, the 10-month level was stimulated with rhTSH. Cervical ultrasound was performed at 10 months, then again 3 years after randomization. Event-free survival was 95.6% in the no-RAI group and 95.9% in the RAI group, for an overall difference of −0.3% (95% CI, −2.7 to −2.2%), which met the study’s non-inferiority criteria. Most of the events (75.7%, 25/33,) were biologic. Secondary analyses also were performed, which found no difference in quality of life, including anxiety and fear of recurrence. The authors concluded that RAI does not confer a benefit over total thyroidectomy alone for these low-risk patients.

## Prophylactic Central Neck Dissection

Central neck dissection/lymphadenectomy (CND) should be performed for clinically evident or pathologically proven metastatic disease in cervical lymph node compartment levels 6 or 7.^1^ These patients would not be considered low risk. In contrast, CND is no longer routinely recommended by the ATA for clinically low-risk disease.^1^ An ATA-sponsored design and feasibility study that examined the potential of a randomized clinical trial to assess the potential for prophylactic CND to reduce recurrence rates in low-risk disease found that prohibitively large sample sizes would be required to demonstrate any difference in outcomes.^27^

However, two smaller studies examined the effects of prophylactic CND on surgical outcomes. Sippel et al.^5^ performed a randomized trial comparing total thyroidectomy with and without CND for patients with clinically node-negative biopsy-proven PTC ≥1 cm in size. In this study, 61 patients were randomized, 30 to thyroidectomy alone and 31 to thyroidectomy plus CND. The primary outcome of the study was the rate of post-surgical hypoparathyroidism, defined as parathyroid hormone (PTH) smaller than 10 pg/mL postoperatively. The groups did not differ in the rate of postoperative hypoparathyroidism, laryngeal nerve palsy, or excellent response to treatment (by ATA criteria^1^). The patients also completed four HRQOL survey instruments, and the researchers found no differences between the treatment groups. In the prophylactic CND group, 27.6% of the patients had positive lymph nodes compared with 10.0% (incidentally removed with the thyroid) of the patients in the thyroidectomy-alone group (*P* = 0.10). Data on cancer recurrence were not provided because the study was underpowered to assess this outcome.

Ahn et al.^6^ performed a similar randomized study in South Korea. The patients scheduled to undergo total thyroidectomy for cT1–2 cN0 PTC between April 2015 and November 2017 were randomized to either total thyroidectomy alone or total thyroidectomy with CND. Of 112 randomized patients, 101 were included in the final analysis, 50 in the thyroidectomy-alone group, and 51 in the CND group. The demographics and tumor characteristics were similar between the groups. These authors found that the rates of lymphatic metastases were similar to those reported by Sippel et al.^5^: 27.5% in the prophylactic CND group and 6.0% in the thyroidectomy-alone group (*P* < 0.05). There were similar rates of “surgical completeness,” defined by the authors as negative postoperative ultrasound and either stimulated Tg < 1 ng/mL (if the patient was treated with RAI) or unstimulated Tg < 0.2 ng/mL (no RAI treatment): 84.0% for thyroidectomy alone and 86.3% for thyroidectomy plus CND (*P* = 1.00). The patients were followed for a mean period of 46.6 months, and no patients experienced structural recurrence in either group. One patient in each group experienced biochemical recurrence. Complication rates, including voice symptoms and hypoparathyroidism, did not differ between the groups. The authors concluded that although there was no difference in complications, prophylactic CND did not confer a benefit in terms of response to treatment or recurrence and may not be necessary for patients with cT1–2 cN0 disease.

These studies, although limited by small sample sizes, support the ATA’s recommendation against routine prophylactic CND for patients with low-risk DTC.

## Active Surveillance and Ablative Techniques

### Active Surveillance

Arguably one of the most impactful studies in the management of patients with low-risk thyroid cancers in the past several decades was an observational study of patients managed with active surveillance alone for treatment of papillary thyroid microcarcinomas (PTmC). The study, published by Ito et al.^28^ in 2003, described the development of an active surveillance program at their institution in Japan and reported patient outcomes. The original study was a retrospective analysis of a self-selected cohort of patients with PTmC who were offered either upfront surgery (thyroid lobectomy or total thyroidectomy with or without lymphadenectomy) or active surveillance with serial ultrasounds every 6 months. Patients were excluded from surveillance if their tumors were close to the trachea or recurrent laryngeal nerve, if the tumors had evidence of high-grade malignancy on biopsy, or if there was suspicion for lymphatic metastases. Surgery was recommended to patients in the active surveillance group if tumor growth was more than 3 mm or there was evidence of additional tumor foci or lymphatic metastases.

The inclusion criteria was met by 732 patients during the study period from 1993 to 2001, with 162 of these patients initially choosing surveillance, and 56 (34.5%) ultimately undergoing surgery. However, most of the patients (83.9%, 47/56) who eventually had surgery after initially selecting active surveillance did so by choice, not because of disease progression. In fact, more than 70% of the surveillance group had no increase in tumor size at any time point during the study, with a mean follow-up time of 46.5 months. On ultrasound, 11 patients (6.8%) in the surveillance group showed suspicious lymph nodes, and 30 patients (18.5%) exhibited suspicion of multifocal thyroid cancer. The authors concluded that a low and acceptable rate of disease progression was evident during surveillance for patients with PTmC, and that it was therefore an appropriate alternate management strategy.

The same group published a follow-up study in 2010, adding to the original cohort of patients.^29^ The expanded study included 340 patients who initially chose surveillance and 1059 patients who underwent upfront surgery. The mean follow-up period was 74 months for the surveillance group and 76 months for the surgery group. Of the 340 patients who chose surveillance, 10.6% ultimately had evidence of disease progression during the study (either tumor growth or new suspicious lymphadenopathy). In the surveillance group, 109 patients converted to surgery, 87 (79.8%) of whom had no evidence of progressive disease at the time they chose surgery. None of the 109 patients who initially chose surveillance but subsequently underwent surgery experienced recurrent cancer postoperatively.

These overlapping case series were subject to significant selection bias, and to date, the available evidence for the comparative safety of active surveillance versus upfront surgery is limited by clinical heterogeneity and potential confounding.^7^ More research is needed to better define the relative risks of nonoperative management versus surgery for low-risk differentiated thyroid cancers, especially those larger than 1 cm.

### Ablative Techniques

Several techniques for ablating tumors have been adapted for use in treating thyroid cancer, including ethanol injection and microwave, laser, and radiofrequency ablation (RFA).^30^ Of these, RFA has gained the most traction.^8,30^ Usually performed under ultrasound guidance, RFA involves inserting a probe that emits radio waves into the target tumor, heating the tissue to destroy it.^30^

In one of the largest case series to date, a group in China analyzed data from 1613 patients treated with RFA for low-risk DTC smaller than 2 cm between 2014 and 2020.^31^ They found that during a mean follow-up period of 58.5 months, 4.3% of the patients experienced either persistent or recurrent thyroid cancer. Recurrence was more common after RFA of larger tumors, multifocal tumors, and subcapsular tumors (within 2 mm of the thyroid capsule). At the final follow-up evaluation, 85.3% of the patients had complete tumor disappearance on ultrasound. The rates of post-procedure hematoma (0.9%) and dysphonia lasting longer than 1 month (0.4%) were similar to historical rates after thyroid lobectomy, although no comparison surgical or surveillance cohort was included in the study.

A recent meta-analysis compared thyroidectomy, active surveillance, and RFA, microwave, and laser ablation techniques.^32^ The authors included 13 studies, the majority of which were from Asia, and noted that five studies had a “high” risk of bias and the remaining eight studies had “unclear” risk of bias, mostly due to selection bias and inadequate follow-up evaluation. They found no difference in disease progression/recurrence or mortality by treatment approach, but noted that the patients managed with active surveillance had higher HRQOL scores than those treated with surgery (no direct comparison was made between surveillance and ablation). Thus, it remains unclear what, if any, HRQOL and procedural risk-related benefits ablation may have over surgery and what oncologic benefits it may have over surveillance alone (Table 3).

**Table 3:** Relative advantages and disadvantages of thyroidectomy, active surveillance, and ablative techniques in the treatment of low-risk differentiated thyroid cancers

	Thyroidectomy	Ablation	Active surveillance
Pathology	Able to obtain tumor pathologic information such as tumor subtype, extrathyroidal extension, and angioinvasion	No pathologic information available beyond biopsy	No pathologic information available beyond biopsy
Complications	Dysphonia	Dysphonia	Tumor progression
	Bleeding/hematoma	Bleeding/hematoma	High crossover-to-surgery rate (~1/3 patients), potentially due to patient anxiety
	Hypothyroidism	Capsule rupture	
Recurrence or progression	Low rate of disease recurrence	Low rate of disease persistence or recurrence in highly selected case series	Higher rates of disease progression although still low; risk of selection bias is high
Quality of life	Possibly largest impact on quality of life, including potential need for lifelong thyroid hormone supplementation	Unclear impact on long-term quality of life	Possibly better quality of life; risk of selection bias is high.

## Conclusions

The surgical management of low-risk differentiated thyroid cancer continues to evolve, but has generally shifted away from total thyroidectomy with central neck dissection to lobectomy alone without adjuvant RAI. In the future, ablative techniques and active surveillance may play an increasingly important role in the treatment of small, low-risk thyroid tumors.
